# Evaluation of housefly (*Musca domestica*) larvae meal and frass as alternative protein sources in late-phase laying hens: Effects on ileal digestibility and energy utilization

**DOI:** 10.1016/j.psj.2026.107904

**Published:** 2026-10-05

**Authors:** Abolfazl Salehizadeh, Mehran Torki, Maryam Darbemamieh, Seyed Davood Sharifi

**Affiliations:** a Animal Science Department, College of Agriculture and Natural Resources, Razi University, Kermanshah, 6715685423, Iran; b Department of Plant Protection, College of Agriculture and Natural Resources, Razi University, Kermanshah, Iran; c Department of Animal and Poultry Science, Faculty of Agricultural Technology, University of Tehran, Pakdasht, Iran

**Keywords:** Apparent ileal digestibility, Housefly frass, Housefly larvae meal, Late-phase laying hens, Synbiotic-enzyme supplementation

## Abstract

The present study evaluated the effects of housefly (Musca domestica) larvae meal (HFLM) and housefly larvae plus frass (HFLF), with or without a commercial synbiotic-enzyme supplement, on apparent ileal nutrient digestibility and apparent ileal digestible energy (AIDE) in Lohmann LSL-Lite laying hens. Two 10-week feeding trials were conducted, each involving 192 hens at 56 weeks of age, using a 4 × 2 factorial design with four inclusion levels (0, 5, 10, and 15%) of HFLM or HFLF and two synbiotic-enzyme supplementation levels (0 and 0.05%). In Experiment 1, HFLM inclusion did not affect nutrient digestibility or AIDE (*P* > 0.05), and no HFLM × synbiotic-enzyme interactions were observed. Synbiotic-enzyme supplementation reduced ash digestibility by 24.1% (42.20 vs. 55.63%; *P* = 0.0312), whereas it increased nitrogen-free extract digestibility (NFE) by 37.1% (72.81 vs. 53.09%; *P* = 0.0099) and AIDE by 23.2% (2836.2 vs. 2301.5 kcal/kg; *P* = 0.0240). In Experiment 2, significant HFLF × synbiotic-enzyme interactions were detected for crude protein (CP), ether extract (EE), ash, NFE, and AIDE (P ≤ 0.0401). The 10% HFLF diet supplemented with 0.05% synbiotic-enzyme produced the highest CP (87.49%), EE (79.15%), ash (70.72%), and NFE (87.49%) digestibility values and AIDE (3402.29 kcal/kg), representing increases of 42.3%, 99.8%, 69.6%, 116.8%, and 96.1%, respectively, relative to the corresponding lowest observed values. The lowest CP, NFE, and AIDE values were observed with 5% HFLF without supplementation, whereas the lowest EE and ash digestibility occurred with 15% HFLF with and without supplementation, respectively. HFLF inclusion also affected crude fiber (*P* = 0.0100) and calcium (*P* = 0.0166) digestibility, which increased by 36.4% and 52.4%, respectively, at 10% HFLF compared with the control. Overall, under the conditions of the present study, HFLM inclusion up to 15% did not adversely affect apparent ileal nutrient digestibility or energy utilization. The responses to HFLF varied with synbiotic-enzyme supplementation, with 10% HFLF plus 0.05% supplementation showing favorable nutrient digestibility and energy utilization responses.

## Introduction

The continued expansion of the global poultry industry has intensified dependence on traditional protein ingredients such as soybean meal and fishmeal, leading to increasing concerns over feed costs, environmental sustainability, and competition with human food resources (Kazemi and Valizadeh, 2025). Consequently, identifying sustainable alternative protein ingredients has become a major priority in poultry nutrition. Aligned with circular bioeconomy principles, insects have gained recognition as sustainable feed resources because they can bioconvert organic waste streams into high-quality biomass, thereby improving resource efficiency and minimizing environmental burdens (Sedgh-Gooya et al., 2021; Kazemi and Valizadeh, 2025; Kawasaki et al., 2026).

Among the emerging alternative protein ingredients, housefly (Musca domestica) larvae meal (**HFLM**) has gained considerable interest because of its short production cycle, high crude protein concentration (**CP**, 40–65%), and well-balanced amino acid and fatty acid composition (Atteh and Adeyoyin, 2021; Salehizadeh et al., 2025a). In addition to larvae meal, insect production generates frass, a coproduct consisting of insect excreta, residual substrate, and exuviae (Tóth et al., 2025). Incorporating frass with larvae meal (**HFLF**) represents a sustainable zero-waste strategy that reduces processing costs while supplying additional minerals and chitin-derived bioactive compounds with potential prebiotic properties (Amorim et al., 2024; Tóth et al., 2025).

Despite these advantages, the feeding value of insect-derived ingredients is largely determined by the extent to which their nutrients can be digested and utilized. Chitin, the principal structural polysaccharide of the insect exoskeleton, behaves as an insoluble dietary fiber that can encapsulate nutrients and reduce their accessibility to endogenous digestive enzymes, thereby impairing nutrient digestibility and energy utilization (Gasco et al., 2019; Salahuddin et al., 2024; Abenaim and Conti, 2025). Because poultry possess limited endogenous chitinolytic activity, these limitations are expected to be more pronounced in HFLF than in purified HFLM owing to its higher content of exuviae and structural fiber (Koh and Iwamae, 2013; Salahuddin et al., 2024). Consequently, HFLF represents a trade-off between sustainability and nutritional efficiency, offering economic and environmental advantages while potentially reducing nutrient utilization.

Dietary supplementation with synbiotics and exogenous enzymes has emerged as a promising strategy to overcome these limitations. Such additives enhance nutrient utilization by degrading structural polysaccharides, increasing nutrient accessibility, modulating the intestinal microbiota, and improving gut integrity (Alagawany et al., 2018; Zhang et al., 2020; Mohammadi et al., 2025; Yalveh et al., 2026). These effects may be particularly beneficial in older laying hens, where age-related reductions in digestive enzyme secretion, intestinal integrity, and absorptive efficiency limit the effective digestion and utilization of fiber-rich diets (Lee et al., 2025; Qiang et al., 2025).

Despite the growing interest in insect meals, data regarding their influence on apparent ileal nutrient digestibility and energy utilization in aging laying hens remain scarce. Furthermore, the comparative efficacy of purified HFLM versus HFLF, and the potential of synbiotic-enzyme supplementation to mitigate chitin-induced digestive challenges, has not been systematically investigated. Accordingly, this study was conducted to evaluate the impact of supplementing the diets of 56-week-old laying hens with HFLM and HFLF, administered individually or together with the synbiotic–enzyme additive, on apparent ileal nutrient digestibility and apparent ileal digestible energy (**AIDE**). We hypothesized that the increased chitin and fiber content of HFLF would depress energy and nutrient utilization, and that synbiotic-enzyme supplementation would mitigate these adverse effects by enhancing intestinal functionality and nutrient availability.

## Materials and methods

### Ethics statement

All animal experiments were performed in accordance with institutional guidelines for laboratory animal welfare and were formally approved by the Razi University Animal Ethics Committee in Kermanshah, Iran (Approval No. IR.RAZI.AEC.1404.014).

### Experimental design and diets

To assess the effects of housefly-derived feed ingredients on nutrient utilization, two independent 10-week feeding studies were conducted using 56-week-old Lohmann LSL-Lite laying hens. The diets were formulated with or without a multi-component feed additive (Rayabold), and its effects on apparent ileal digestibility (**AID**) of nutrients and AIDE were evaluated. Both experiments were conducted under identical management conditions and followed the same experimental design.

Each experiment involved 192 healthy laying hens randomly assigned to a completely randomized 4 × 2 factorial design. The factors consisted of four inclusion levels of the test ingredient (0, 5, 10, and 15%) and two dietary levels of Rayabold (0 and 0.05%). Each treatment was replicated four times, with six hens housed per cage (45 × 45 × 45 cm), resulting in 24 hens per treatment. The number of replicates was determined based on the expected variability and biologically relevant differences in nutrient digestibility reported in previous poultry studies. Four independent cage replicates per treatment were considered adequate to detect moderate-to-large treatment effects at a significance level of α = 0.05. Across the two experiments, a total of 64 experimental cages and 384 hens were used.

Experiment 1 evaluated HFLM, whereas Experiment 2 evaluated HFLF. The birds were reared under standardized environmental conditions, with a 16:8 h light-dark cycle, room temperature maintained at 18–21°C, and relative humidity between 30 and 40%. Feed was offered at approximately 120 g per hen per day, and drinking water was provided ad libitum throughout the study.

Housefly larvae meal and HFLF were obtained from Fardad Kian Fartak Company (Iran). Prior to diet formulation, representative samples of HFLM and HFLF were subjected to chemical analysis to determine their nutrient composition. Dry matter (**DM**), CP (Kjeldahl nitrogen × 6.25), ether extract (**EE**), crude fiber (**CF**), ash, calcium (**Ca**), and phosphorus (**P**) were determined according to the official methods of the AOAC (2005). Carbohydrate concentration was estimated by difference. The amino acid profile of HFLM was determined using high-performance liquid chromatography (**HPLC**) following acid hydrolysis. Methionine and cysteine were quantified after performic acid oxidation, whereas tryptophan was analyzed following alkaline hydrolysis. The proximate composition of HFLM and HFLF, along with the amino acid composition of HFLM, is summarized in Table 1, Table 2, respectively.

**Table 1:** Chemical composition of housefly larvae meal (HFLM) and housefly larvae plus frass (HFLF).

Parameter	HFLM	HFLF
Dry matter (%)	93.76	90.81
Crude protein (%)	53.49	22.41
Crude fat (%)	20.99	1.80
Crude fiber (%)	8.59	16.67
Carbohydrates (%)	1.86	34.20
Digestible protein (%)	98.27	93.10
Metabolizable energy (kcal/kg)	3,460	2,460
Calcium (%)	12.1	28.2
Phosphorus (%)	1.47	1.97
Ash (%)	5.77	7.98

**Table 2:** Amino acid composition of housefly larvae meal (HFLM) expressed as a percentage of feed protein.

Amino Acid	Content (% of crude protein)
Aspartic acid	0.35
Glutamic acid + Glutamine	5.01
Asparagine	0.24
Serine	1.82
Histidine	2.14
Glycine	9.11
Threonine	3.17
Arginine	3.89
Alanine	11.77
Tyrosine	1.77
Cysteine	ND
Valine	1.28
Methionine	4.02
Tryptophan	0.19
Phenylalanine	3.23
Isoleucine	0.58
Leucine	1.63
Lysine	0.21

All diets were formulated according to the nutrient specifications for Lohmann LSL-Lite laying hens established by Lohmann Breeders (2020), ensuring that all nutritional requirements were met or exceeded. Details of the ingredient composition and calculated nutrient contents of the experimental diets are presented in Table 3.

**Table 3:** Composition of ingredients and calculated nutrient contents of experimental diets containing graded concentrations of housefly larvae meal (HFLM) or a housefly larvae–frass mixture (HFLF), with or without synbiotic-enzyme supplementation (Rayabold).

Diets							
Ingredients (%)	Control	HFLM5	HFLM10	HFLM15	HFLF5	HFLF10	HFLF15
Corn	59.20	57.99	56.89	55.58	52.5	49.69	45.91
Soybean meal	27.50	21.00	13.76	7.12	27.36	25.98	24.78
Wheat bran	-	3.00	6.00	6.50	-	-	-
Calcium carbonate	10.80	10.92	11.01	11.07	10.84	10.88	10.91
HFLM or HFLF	-	5.00	10.00	15.00	5	10	15
Bentonite	-	-	0.50	3.00	0.79	-	-
Dicalcium phosphate	1.00	0.66	0.32	0.02	0.97	0.9	0.84
Multi mix^a^	0.50	0.50	0.50	0.50	0.5	0.5	0.5
Rayabold^b^	-	0.05	0.05	0.05	0.05	0.05	0.05
Binder toxin	0.10	0.10	0.10	0.10	0.1	0.1	0.1
DL-methionine	0.15	0.08	-	-	0.19	0.2	0.21
L-lysine	-	-	0.17	0.36	-	-	-
Salt	0.20	0.20	0.20	0.20	0.2	0.2	0.2
Oil	0.50	0.50	0.50	0.50	1.5	1.5	1.5
***Calculated composition***							
ME (kcal/kg)	2700	2700	2700	2700	2700	2670	2670
Crude protein (%)	17.00	17.00	17.00	17.00	17	17	17
Crude fiber (%)	3.14	2.90	2.68	2.23	3.14	3.56	4.06
Calcium (%)	4.20	4.20	4.20	4.20	4.2	4.2	4.2
Available phosphorus (%)	0.42	0.42	0.42	0.42	0.42	0.42	0.42
Lysine (%)	0.81	0.68	0.65	0.65	0.81	0.81	0.79
Methionine + Cysteine (%)	0.63	0.66	0.66	0.76	0.63	0.66	0.66
Threonine (%)	0.60	0.67	0.73	0.79	0.6	0.59	0.58
Tryptophan (%)	0.20	0.18	0.15	0.12	0.203	0.2	0.19
Arginine (%)	1.10	1.12	1.11	1.1	1.1	1.09	1.06
Isoleucine (%)	0.69	0.61	0.51	0.41	0.69	0.66	0.63
Valine (%)	0.77	0.73	0.66	0.6	0.77	0.74	0.7

a Vitamin mixture per 0.25 kg/100 kg of diet: Vitamin A, 7,700,000 IU; Vitamin D3, 3,300,000 IU; Vitamin E, 6600 mg; VitaminK3, 550 mg; thiamine,2200 mg; riboflavin, 4400 mg; Vitamin B6, 4400 mg; capantothenate, 550 mg; nicotinic acid, 200 mg; folic acid, 110 mg; choline chloride, 275,000 mg; biotin,55 mg; Vitamin B12, 8.8 mg. Mineral mixture per 0.25 kg/100 kg of diet: Mn, 66,000 mg; Zn, 66,000 mg; Fe, 33,000 mg; Cu, 8,800 mg; Se, 300 mg.

b Rayabold was included at either 0 or 0.05% of the diet. It is a commercial multi-component feed additive containing probiotics, prebiotics, and exogenous enzymes. Because the control diet had the same basal ingredient composition with or without Rayabold, it is presented once in the table to avoid repetition.

Diets were supplemented with either 0 or 0.05% Rayabold, a commercial product manufactured by Fardad Kian Fartak Company (Iran). The additive is a synbiotic and enzymatic blend providing 1 × 10⁹ CFU/g of probiotic microorganisms, including L. plantarum, L. delbrueckii, E. faecium, L. casei, L. rhamnosus, B. bifidum, B. coagulans, B. subtilis, B. licheniformis, S. salivarius, and L. acidophilus. It also contains prebiotic substrates, including autolyzed yeast, mannan oligosaccharides, beta-glucan, and inulin, together with a specific enzyme complex. Per kilogram of product, the enzyme complex provides 1,200,000 U of protease, 75,000 U of lipase, 30,000 U of glucose oxidase, 30,000 U of alpha-amylase, 20,000 U each of hemicellulase, beta-amylase, xylanase, and beta-glucanase, and 10,000 U of phytase.

### Determination of nutrient digestibility and energy

Following the 10-week experimental period, the AID of nutrients and AIDE were evaluated using acid-insoluble ash (**AIA**) as an indigestible marker.

### Experimental procedure

For the apparent ileal digestibility assay, one hen was randomly selected from each replicate cage. Thus, 32 hens were sampled per trial, giving a total of 64 sampled hens across the two trials. The replicate cage was considered the experimental unit, while the individually selected hen served as the sampling unit for ileal digesta collection. Selected hens were individually housed under the same environmental and feeding conditions as those maintained during the experimental period. Digestibility measurements were conducted over four days, comprising a 1-day adaptation period followed by a 3-day ileal digesta collection period. To facilitate digestibility determination, all experimental diets were supplemented with 5 g/kg AIA as an indigestible marker.

At the conclusion of the collection period, birds were humanely euthanized by cervical dislocation. Ileal digesta samples were collected from the intestinal segment extending from Meckel's diverticulum to approximately 2 cm proximal to the ileocecal junction. The digesta were gently flushed with distilled water, collected individually, immediately frozen at −20°C, and subsequently dried in a forced-air oven before chemical analysis.

### Chemical analyses and energy estimation

Representative samples of diets and ileal digesta were analyzed for CP, EE, CF, ash, nitrogen-free extract (**NFE**), Ca, P, gross energy (**GE**), and AIA following AOAC (2005) procedures.

NFE was estimated by difference:

NFE(%)=100−(CP+EE+CF+Ash)

The GE content of the diets and ileal digesta was estimated by multiplying the analyzed nutrient concentrations by their respective energy coefficients. The GE was calculated as the sum of the energy contributed by CP, CF, EE, and NFE according to the following equation:

GE(kcal/kg)=(CP×5.7)+(CF×4.1)+(EE×9.4)+(NFE×4.1)

The AID of nutrients was determined using the formula:

$$\text{AI}D_{i^{\left(\%\right)}}=\left\{1-\left[\left(\frac{AIA_{d}}{AIA_{i}}\right)\times \left(\frac{N_{i}}{N_{d}}\right)\right]\right\}\times \;100$$

where (AIA_d_) and (AIA_i_) represent the concentrations of acid-insoluble ash in the diet and ileal digesta, respectively, and (N_i)_ and (N_d_) represent the concentrations of the nutrient of interest in the diet and ileal digesta, respectively.

AIDE (kcal/kg) was calculated using the indicator method to account for energy absorption up to the terminal ileum:

$$AIDE\;\left(\frac{kcal}{kg}\right)=\;GE_{d}-\left[GE_{i}\times \;\left(\frac{AIA_{d}}{AIA_{i}}\right)\right]$$

Where d denotes the diet and i denotes the ileal digesta.

### Statistical analysis

The datasets generated from Experiments 1 and 2 were analyzed separately. Before conducting the statistical analyses, the normality of the residuals was assessed using the Shapiro–Wilk test. For each experiment, a two-factor analysis of variance (**ANOVA**) was performed using the General Linear Model (**GLM**) procedure in SAS software (Version 9.4; SAS, 2015). Treatments were arranged in a completely randomized 4 × 2 factorial design, consisting of four inclusion levels of the respective test ingredient (HFLM in Experiment 1 or HFLF in Experiment 2) and two levels of synbiotic-enzyme supplementation (0 and 0.05%). The fixed effects included dietary inclusion level, synbiotic-enzyme supplementation, and their interaction. The data were fitted to the following statistical model:

$$Y_{ijk}\;=\;\mu \;+\;HF_{i}\;+\;R_{j}\;+\;{\left(HFR\right)}_{ij}\;+\;e_{ijk}$$

where *Y_ijk_* is the observed response, *μ* is the overall mean, *HF_i_* represents the effect of dietary HFLM or HFLF inclusion level, *R_j_* denotes the effect of synbiotic-enzyme supplementation, *HFR_ij_* is the interaction between the two factors, and *e_ijk_* is the residual error. When the ANOVA indicated significant treatment effects, pairwise comparisons of treatment means were carried out using Tukey's post hoc test. Significance was determined using a threshold of P ≤ 0.05.

## Results

The effects of dietary HFLM and synbiotic-enzyme supplementation on the AID of nutrients and AIDE in 56-week-old laying hens are presented in Table 4. The interaction between dietary HFLM inclusion and synbiotic-enzyme supplementation had no significant effect on the AID of CP, CF, EE, P, Ca, ash, or NFE, or on AIDE (*P* > 0.05). Similarly, HFLM inclusion at levels ranging from 0 to 15% did not significantly affect any of the measured digestibility parameters (*P* > 0.05). Synbiotic-enzyme supplementation did not significantly affect the AID of CP, CF, EE, P, or Ca (*P* > 0.05). However, it significantly reduced ash digestibility from 55.63 to 42.20% (24.1% decrease**,** *P* = 0.0312), while increasing NFE digestibility from 53.09 to 72.81% (37.1% increase, *P* = 0.0099) and AIDE from 2301.5 to 2836.2 kcal/kg (23.2% increase, *P* = 0.0240).

**Table 4:** Effects of dietary HFLM and synbiotic-enzyme supplementation on nutrient digestibility coefficients and AIDE in 56-week-old laying hens.

Treatments			CP (%)	CF (%)	EE (%)	P (%)	Ca (%)	Ash (%)	NFE (%)	AIDE (kcal/kg)
Main effects										
HFLM (%)										
0			74.10	56.22	56.03	50.53	61.69	42.71	65.49	2614.8
5			71.53	55.80	57.68	51.41	50.81	47.96	54.87	2294.7
10			85.00	58.62	57.44	55.88	60.45	53.72	70.25	2883.9
15			77.63	62.77	60.55	56.57	54.23	51.28	61.50	2480.8
Synbiotic-enzyme (%)										
0			76.16	59.09	53.03	53.98	57.46	55.63^a^	53.09^b^	2301.5^b^
0.05			77.97	61.67	62.75	52.98	56.60	42.20^b^	72.81^a^	2836.2^a^
Interactions										
HFLM	Synbiotic-enzyme									
0		0	66.24	52.35	53.43	49.66	63.43	47.76	53.80	3017.94
5		0	76.48	59.08	58.32	48.48	47.22	58.42	54.03	2235.96
10		0	87.26	51.14	44.50	60.42	59.96	58.32	61.50	3045.72
15		0	78.68	53.79	56.00	57.33	59.24	58.01	52.34	3045.14
0		0.05	81.97	60.08	57.76	51.41	59.94	37.68	77.18	2211.69
5		0.05	66.58	52.51	57.03	54.34	56.19	37.50	55.42	2011.80
10		0.05	82.73	66.09	70.38	49.07	60.94	49.11	78.99	2722.05
15		0.05	80.58	65.75	67.38	55.81	49.21	44.54	79.67	2001.60
SEM			8.51	14.46	16.90	15.58	14.49	13.91	15.81	300.78
P value										
HFLM			0.6107	0.8940	0.9519	0.8989	0.6215	0.5646	0.4554	0.3122
Synbiotic-enzyme			0.0713	0.2498	0.1905	0.8434	0.8854	0.0312	0.0099	0.0240
HFLM × Synbiotic-enzyme			0.0822	0.6313	0.5614	0.8325	0.7512	0.1001	0.3727	0.2138

HFLM = housefly larvae meal; CP = crude protein; CF = crude fiber; EE = ether extract; *P* = phosphorus; Ca = calcium; NFE = nitrogen-free extract; AIDE = apparent ileal digestible energy. SEM = standard error of the mean. Different superscript letters assigned to means within the same column and effect indicates statistically significant differences (P ≤ 0.05).

The effects of dietary HFLF, synbiotic-enzyme supplementation, and their interaction on nutrient AID and AIDE are presented in Table 5. Significant interactions between HFLF inclusion and synbiotic-enzyme supplementation were observed for CP (*P* = 0.0001), EE (*P* = 0.0017), ash (*P* = 0.0401), NFE (*P* = 0.0018), and AIDE (*P* = 0.0002). The combination of 10% HFLF with 0.05% synbiotic-enzyme supplementation resulted in the highest CP (87.49%), EE (79.15%), ash (70.72%), and NFE (87.49%) digestibility, as well as the highest AIDE (3402.29 kcal/kg). Relative to the corresponding lowest observed values, these represented increases of 42.4% for CP (from 61.46 to 87.49%), 99.8% for EE (from 39.63 to 79.15%), 69.6% for ash (from 41.70 to 70.72%), 116.8% for NFE (from 40.35 to 87.49%), and 96.1% for AIDE (from 1734.55 to 3402.29 kcal/kg). The lowest CP digestibility (61.46%), NFE digestibility (40.35%), and AIDE (1734.55 kcal/kg) were observed in hens fed 5% HFLF without synbiotic-enzyme supplementation. The lowest EE digestibility (39.63%) occurred with 15% HFLF plus synbiotic-enzyme supplementation, whereas the lowest ash digestibility (41.70%) occurred with 15% HFLF without supplementation.

**Table 5:** Effect of dietary HFLF and synbiotic-enzyme supplementation on nutrient digestibility coefficients and AIDE in 56-week-old laying hens.

Treatments				CP (%)	CF (%)	EE (%)	P (%)	Ca (%)	Ash (%)	NFE (%)	AIDE (kcal/kg)
Main effects											
HFLF (%)											
0				73.84^c^	46.03^b^	65.50^a^	44.88	49.84^b^	49.27^b^	69.03^b^	2663.41^b^
5				67.80^d^	57.10^ab^	53.67^b^	51.16	56.14^b^	46.26^b^	56.78^c^	2299.46^c^
10				84.30^a^	62.78^a^	69.75^a^	66.18	75.93^a^	63.24^a^	75.87^a^	3001.45^a^
15				77.86^b^	60.51^a^	51.58^b^	49.60	58.76^b^	45.90^b^	68.81^b^	2751.68^b^
Synbiotic-enzyme (%)											
0				75.37	60.40	61.29	57.94	64.68	49.70	59.92^b^	2413.91^b^
0.05				77.16	54.74	59.44	47.67	57.61	52.96	75.58^a^	2953.62^a^
Interactions											
HFLF	Synbiotic-enzyme										
0	0			70.93^e^	56.90	73.12^ab^	61.81	58.42	50.23^bc^	62.39^d^	2419.39^e^
5	0			61.46^f^	59.41	48.18^dc^	57.73	53.28	52.06^bc^	40.35^e^	1734.55^f^
10	0			81.10^bc^	62.57	60.34^bc^	66.80	79.23	55.76^b^	64.24^cd^	2600.59^de^
15	0			83.36^ab^	61.29	63.52^abc^	62.44	61.90	41.70^c^	66.17^cd^	2674.65^cd^
0	0.05			76.74^de^	49.19	57.88^bc^	44.95	44.12	48.62^bc^	75.67^b^	2907.42^b^
5	0.05			72.03^e^	55.56	61.91^bc^	41.31	58.05	42.39^bc^	67.73^cd^	2676.06^cd^
10	0.05			87.49^a^	62.99	79.15^a^	65.55	72.64	70.72^a^	87.49^a^	3402.29^a^
15	0.05			72.36^de^	61.39	39.63^d^	36.76	55.63	50.10^bc^	71.45^bc^	2828.69^bc^
SEM				2.46	6.63	8.79	12.40	11.64	6.58	4.10	115.97
P value											
HFLF				0.0001	0.010	0.0093	0.2059	0.0166	0.0017	0.0001	0.0001
Synbiotic-enzyme				0.1250	0.0662	0.6627	0.1610	0.2855	0.3075	0.0001	0.0001
HFLF × Synbiotic-enzyme				0.0001	0.1710	0.0017	0.5367	0.6594	0.0401	0.0018	0.0002

HFLF = housefly larvae meal + frass; CP = crude protein; CF = crude fiber; EE = ether extract; *P* = phosphorus; Ca = calcium; NFE = nitrogen-free extract; AIDE = apparent ileal digestible energy. SEM = standard error of the mean. Different superscript letters assigned to means within the same column and effect indicates statistically significant differences (P ≤ 0.05).

Dietary HFLF inclusion significantly affected CF (*P* = 0.0100) and Ca (*P* = 0.0166) digestibility. CF digestibility increased from 46.03% at 0% HFLF to 62.78% at 10% HFLF, representing a 36.4% increase, while the 15% HFLF diet yielded 60.51%, a 31.5% increase relative to the control. Calcium digestibility was highest at 10% HFLF (75.93%), representing a 52.4% increase compared with 0% HFLF (49.84%), whereas values at 5 and 15% HFLF were 56.14% and 58.76%, respectively. Neither HFLF inclusion nor synbiotic-enzyme supplementation significantly affected P digestibility (*P* > 0.05).

## Discussion

### Housefly larvae meal and synbiotic-enzyme supplementation

The present study demonstrated that dietary inclusion of HFLM at levels up to 15% did not adversely affect the AID of CP, EE, CF, ash, NFE, Ca, P, or AIDE in 56-week-old laying hens. These findings indicate that HFLM can be incorporated at up to 15% without adversely affecting apparent nutrient digestibility under the conditions of the present study, supporting its potential as an alternative feed ingredient for laying hens (da-Silva et al., 2024; Kawasaki et al., 2026).

The high digestibility of HFLM is consistent with its favorable nutritional profile, including highly digestible protein, a balanced essential amino acid composition, and readily available lipids (Nayab et al., 2025; Ochoa et al., 2025; Kawasaki et al., 2026). Unlike many plant protein feedstuffs, insect meals contain comparatively smaller amounts of anti-nutritional constituents, including phytate and non-starch polysaccharides (**NSPs**), which facilitates improved nutrient utilization (Gasco et al., 2019; Elahi et al., 2022). Although insect meals contain chitin, its concentration at the inclusion levels evaluated was apparently insufficient to impair nutrient utilization. Mature laying hens may also utilize moderate amounts of chitin more efficiently because of their well-developed gastrointestinal tract and endogenous chitinase activity (Lee et al., 2022; Gasco et al., 2023).

These results corroborate earlier reports highlighting the excellent digestibility and nutritional value of HFLM as a poultry feed ingredient (Zuidhof et al., 2003; Hwangbo et al., 2009; Pretorius, 2011; Pieterse and Pretorius, 2014). Earlier studies reported CP digestibility coefficients ranging from approximately 69% to 99%, EE digestibility exceeding 90%, and AME values between 14.2 and 17.9 MJ/kg, confirming that HFLM constitute a highly digestible source of dietary protein and energy. The present results also support previous reports showing that insect meals can replace conventional protein sources without impairing nutrient utilization. Khan et al. (2024) likewise demonstrated that dietary substitution of soybean meal with maggot meal at inclusion rates of up to 30% had no adverse effect on the digestibility of CP, EE, CF, or ash in broilers. Although absolute digestibility coefficients differed between studies, the absence of significant treatment effects was consistent, suggesting that differences were primarily associated with bird type (laying hens vs. broiler chicks), insect meal composition, dietary formulation, inclusion level, and digestibility methodology (ileal versus total tract).Processing method is another important determinant of nutrient utilization. Ajiboye et al. (2022) reported that roasted maggot meal resulted in greater CP and EE digestibility than sun-dried meal, likely due to improved protein denaturation, lipid accessibility, and nutrient preservation. These findings highlight that the nutritive value of insect meals depends on both insect species and post-harvest processing.

The absence of a significant interaction between HFLM and synbiotic-enzyme supplementation across all digestibility indices suggests that the two treatments exerted independent effects on nutrient utilization. This is biologically plausible because HFLM is highly digestible and contains relatively low amounts of structural carbohydrates requiring enzymatic degradation (da-Silva et al., 2024; Nayab et al., 2025). Consequently, the carbohydrases supplied by the synbiotic-enzyme supplement likely acted mainly on the corn–soybean meal fraction rather than the insect meal component (Abbasi Arabshahi et al., 2021; Attia et al., 2022; Yaqoob et al., 2022).

Although synbiotic-enzyme supplementation had little effect on the digestibility of CP, EE, CF, Ca, or P, it significantly improved NFE digestibility and AIDE. This finding is consistent with the established mechanisms by which multi-enzyme and synbiotic additives improve nutrient utilization in cereal-based poultry diets. Corn and soybean meal contain NSPs that can limit starch accessibility, whereas exogenous carbohydrases hydrolyze these polysaccharides, reduce digesta viscosity, and increase starch availability to endogenous enzymes (Abbasi Arabshahi et al., 2021; Jha and Mishra, 2021; Yaqoob et al., 2022). Enhanced carbohydrate digestion consequently improves glucose absorption and dietary energy utilization.

The improvement in AIDE therefore appears to result primarily from enhanced carbohydrate digestion rather than increased protein or lipid utilization. Previous studies have similarly demonstrated enhanced energy utilization in poultry receiving corn–soybean meal diets supplemented with exogenous enzymes (Zou et al., 2013; Alagawany et al., 2018; Alqhtani et al., 2024; Yi et al., 2024; Oketch and Heo, 2026). In addition, the probiotic microorganisms and prebiotic substrates contained in the synbiotic-enzyme supplement may have further enhanced intestinal microbial activity and short-chain fatty acid production, thereby improving gut health and overall nutrient utilization (Al-Khalaifah, 2018).

An unexpected finding was the reduction in ash digestibility following synbiotic-enzyme supplementation despite the improvement in AIDE. Similar responses have been reported following synbiotic supplementation and may reflect altered microbial mineral utilization, changes in mineral solubility, or redistribution of minerals within the intestinal lumen rather than impaired mineral absorption (Gungor and Erener, 2020; Yi et al., 2024; Liu et al., 2026). Because mineral retention, serum mineral concentrations, and bone mineralization were not measured, the biological significance of this response remains uncertain and warrants further investigation.

### Housefly larvae meal plus frass and synbiotic-enzyme supplementation

Unlike HFLM, HFLF exhibited significant interactions with synbiotic-enzyme supplementation for CP, EE, ash, NFE, and AIDE, with the greatest responses observed at 10% HFLF combined with 0.05% synbiotic-enzyme supplementation. These findings indicate that the nutritional value of HFLF depends on both its composition and the digestive environment influenced by the feed additive.

This synergistic response is likely related to the complex composition of insect frass, which comprises undigested feed residues, larval exuviae, beneficial microorganisms, organic minerals, and chitin (Adams and Koutsos, 2024; Hatab et al., 2025). Consequently, the HFLF mixture contains approximately 22% CP, 1.8% EE, and up to 16% CF (Salehizadeh et al., 2025b). The improved digestibility of CP, EE, and NFE following synbiotic-enzyme supplementation likely resulted from the complementary actions of its enzyme and synbiotic components. Carbohydrases reduce structural barriers surrounding encapsulated nutrients, while probiotic microorganisms may enhance intestinal microbial activity and gut function (Krysiak et al., 2021; Musigwa et al., 2021). In addition, partial degradation of chitin may generate chito-oligosaccharides with prebiotic properties, further supporting beneficial microbial populations, although these mechanisms were not directly assessed in the present study (Han et al., 2024; Rangel et al., 2024; Yang et al., 2025).

The corresponding increase in AIDE is therefore likely attributable to improved carbohydrate digestion and nutrient absorption, consistent with previous reports of enzyme–synbiotic supplementation in structurally complex poultry diets (Yaqoob et al., 2022; Bedford et al., 2023).

In the absence of synbiotic-enzyme supplementation, HFLF primarily affected CF and Ca digestibilities, with the 10% inclusion level producing the most favorable response, whereas P digestibility remained unaffected. This response is closely associated with the physical and chemical characteristics of insect frass, whose fiber fraction consists mainly of crystalline chitin and residual plant fiber from the rearing substrate (Adams and Koutsos, 2024; Pascon et al., 2025). At a moderate inclusion level, these insoluble fibers likely stimulated gizzard development and muscular activity, improving feed particle grinding and regulating digesta passage (Jha and Mishra, 2021; Sekh and Karki, 2022). The resulting increase in gastric retention time would enhance exposure to hydrochloric acid and pepsin, promoting Ca solubilization and increasing the availability of Ca²⁺ for absorption in the small intestine (Jha and Mishra, 2021; Penazzi et al., 2021; Belhadj Slimen et al., 2023). This mechanism may explain the highest Ca digestibility observed at 10% HFLF. In contrast, increasing HFLF to 15% without synbiotic-enzyme supplementation may have negated these benefits. Higher concentrations of chitin and fiber may bind Ca within the insoluble matrix, reducing its availability, while the greater ash content may disrupt the dietary Ca-to-P ratio, promoting mineral antagonism and increasing Ca excretion (Agunbiade et al., 2007; Gasco et al., 2023; Rahayu et al., 2023).

The present findings are in agreement with previous reports on the use of insect-derived feed ingredients in poultry nutrition. Bovera et al. (2018) observed that high dietary inclusion of black soldier fly larvae meal in place of soybean meal reduced nutrient digestibility, an effect largely attributed to the elevated chitin content of the insect meal. Likewise, studies on black soldier fly frass have shown that moderate dietary inclusion can be effectively utilized, whereas excessive inclusion may impair nutrient utilization unless diets are carefully formulated (Yildirim-Aksoy et al., 2020; Adams and Koutsos, 2024). Collectively, these studies suggest that insect-derived coproducts have an optimal dietary inclusion range beyond which structural components may begin to limit digestive efficiency.

## Conclusion

The findings of this study indicate that incorporating HFLM into the diets of late-phase laying hens at levels of up to 15% did not adversely affect apparent ileal nutrient digestibility or AIDE under the conditions of the present study, supporting its potential as an alternative protein ingredient. In contrast, supplementation with a synbiotic-enzyme additive appeared to improve the utilization of HFLF. The combination of 10% HFLF and 0.05% synbiotic-enzyme supplementation was associated with favorable nutrient digestibility and energy utilization responses; however, these findings should be interpreted cautiously because of the limited replication. Collectively, the results suggest that HFLM can be incorporated without additional digestive support, whereas synbiotic-enzyme supplementation may enhance the utilization of HFLF. These findings highlight the importance of considering the physicochemical characteristics of insect-derived feed ingredients when formulating poultry diets.

Further research with greater replication is needed to confirm these observations and to investigate the mechanisms underlying the responses, particularly intestinal microbial ecology, chitin degradation, digestive enzyme activity, and nutrient transport, as well as responses to these ingredients under different dietary compositions and feeding systems.

## Ethics declaration

### Animal subject

This study was conducted in accordance with the ARRIVE (Animal Research: Reporting of In Vivo Experiments) guidelines. This study was approved by the Razi University Animal Ethics Committee in Kermanshah, Iran. (Approval No. IR.RAZI.AEC.1404.014).

## Author contributions

Abolfazl Salehizadeh: 1. Substantial Contributions: The acquisition, analysis, or interpretation of data. 2. Drafting or Reviewing: Writing the manuscript draft 3. Final Approval: Reviewing and explicitly approving the exact, final version of the manuscript before it goes to press. 4. Accountability: Agreeing to be personally and publicly responsible for all aspects of the work, ensuring that any questions regarding the accuracy or integrity of the research are thoroughly investigated and resolved. Mehran Torki: 1. Substantial Contributions: Substantive input into the conception or design of the work, and the interpretation of data. 2. Drafting or Reviewing: Reviewing it critically for crucial intellectual content. 3. Final Approval: Reviewing and explicitly approving the exact, final version of the manuscript before it goes to press. 4. Accountability: Agreeing to be personally and publicly responsible for all aspects of the work, ensuring that any questions regarding the accuracy or integrity of the research are thoroughly investigated and resolved. Maryam Darbemamieh: 1. Substantial Contributions: Substantive input into the conception or design of the work, and the interpretation of data. 2. Drafting or Reviewing: Reviewing it critically for crucial intellectual content. 3. Final Approval: Reviewing and explicitly approving the exact, final version of the manuscript before it goes to press. 4. Accountability: Agreeing to be personally and publicly responsible for all aspects of the work, ensuring that any questions regarding the accuracy or integrity of the research are thoroughly investigated and resolved. Seyed Davood Sharifi: 1. Substantial Contributions: Substantive input into the conception or design of the work, and the interpretation of data. 2. Drafting or Reviewing: Reviewing it critically for crucial intellectual content. 3. Final Approval: Reviewing and explicitly approving the exact, final version of the manuscript before it goes to press. 4. Accountability: Agreeing to be personally and publicly responsible for all aspects of the work, ensuring that any questions regarding the accuracy or integrity of the research are thoroughly investigated and resolved.

## Disclosures

The authors declare that they have no known competing financial interests or personal relationships that could have appeared to influence the work reported in this paper.

## References

[bib0001] Abbasi Arabshahi H., Ghasemi H.A., Hajkhodadadi I., Khaltabadi Farahani A.H. (2021). Effects of multicarbohydrase and butyrate glycerides on productive performance, nutrient digestibility, gut morphology, and ileal microbiota in late-phase laying hens fed corn- or wheat-based diets. Poult. Sci..

[bib0002] Abenaim L., Conti B. (2025). Harnessing chitin from edible insects for livestock nutrition. Insects.

[bib0003] Adams D.S., Koutsos E.A. (2024). Applied research note: black soldier fly larvae frass is an effective feed ingredient for broiler diets. J. Appl. Poult. Res..

[bib0004] Agunbiade J.A., Adeyemi O.A., Ashiru O.M., Awojobi H.A., Taiwo A.A., Oke D.B., Adekunmisi A.A. (2007). Replacement of fish meal with maggot meal in cassava-based layers' diets. J. Poult. Sci..

[bib0005] Ajiboye O.O., Ademola S.G., Arasi K.K., Shittu M.D., Akinwumi A.O., Togun M.E. (2022). Evaluation of differently processed maggot (*Musca domestica*) meal as a replacement for fishmeal in broiler diets. Acta Univ. Agric. Silvic. Mendel. Brun..

[bib0007] Al-Khalaifah H.S. (2018). Benefits of probiotics and/or prebiotics for antibiotic-reduced poultry. Poult. Sci..

[bib0006] Alagawany M., Elnesr S.S., Farag M.R. (2018). The role of exogenous enzymes in promoting growth and improving nutrient digestibility in poultry. Iran. J. Vet. Res..

[bib0008] Alqhtani A.H., Al Sulaiman A.R., Alharthi A.S., Abudabos A.E. (2024). Growth performance and nutrient digestibility of broilers fed low-energy corn-soybean meal-based diets supplemented with an exogenous enzyme cocktail as a combined activity. Rev. Bras. Zootec..

[bib0009] Amorim H.C., Ashworth A.J., Arsi K., Rojas M.G., Morales-Ramos J.A., Donoghue A., Robinson K. (2024). Insect frass composition and potential use as an organic fertilizer in circular economies. J. Econ. Entomol..

[bib0010] AOAC (2005). Off. Methods Anal..

[bib0011] Atteh J.O., Adeyoyin D.D. (2021). Effects of replacing dietary fishmeal with maggots on performance and nutrient retention of laying hens. Niger. J. Anim. Prod..

[bib0012] Attia Y.A., Al-Khalaifah H.S., Alqhtani A.H., Abd El-Hamid H.S., Alyileili S.R., El-Hamid A.E.E.A., Bovera F., El-Shafey A.A. (2022). The impact of multi-enzyme fortification on growth performance, intestinal morphology, nutrient digestibility, and meat quality of broiler chickens fed a standard or low-density diet. Front. Vet. Sci..

[bib0013] Bedford M.R., Svihus B., Cowieson A.J. (2024). Dietary fibre effects and the interplay with exogenous carbohydrases in poultry nutrition. Anim. Nutr..

[bib0014] Belhadj Slimen I., Yerou H., Ben Larbi M., M'Hamdi N., Najar T. (2023). Insects as an alternative protein source for poultry nutrition: a review. Front. Vet. Sci..

[bib0015] Bovera F., Loponte R., Pero M.E., Cutrignelli M.I., Calabrò S., Musco N., Vassalotti G., Panettieri V., Lombardi P., Piccolo G., Di Meo C. (2018). Laying performance, blood profiles, nutrient digestibility and inner organs traits of hens fed an insect meal from *Hermetia illucens* larvae. Res. Vet. Sci..

[bib0033] L. Breeders. 2020. LSL-Lite Layers-Management Guide. Lohmann, Cuxhaven, Germany. https://lohmann-breeders.com/media/strains/cage/management/LOHMANN-LSL-Lite-Cage-1.pdf.

[bib0016] da-Silva W.C., Silva É.B.R., Silva J.A.R., Martorano L.G., Belo T.S., Sousa C.E.L., Camargo-Júnior R.N.C., Andrade R.L., Santos A.G.S., Carvalho K.C., Lobato A.S.M., Rodrigues T.C.G.C., Araújo C.V., Lima J.S., Neves K.A.L., Silva L.K.X., Lourenço-Júnior J.B. (2024). Nutritional value of the larvae of the black soldier fly (*Hermetia illucens*) and the house fly (*Musca domestica*) as a food alternative for farm animals—A systematic review. Insects.

[bib0017] Elahi U., Xu C.C., Wang J., Lin J., Wu S.G., Zhang H.J., Qi G.H. (2022). Insect meal as a feed ingredient for poultry. Anim. Biosci..

[bib0018] Gasco L., Biasato I., Dabbou S., Schiavone A., Gai F. (2019). Animals fed insect-based diets: state-of-the-art on digestibility, performance and product quality. Animals (Basel).

[bib0019] Gasco L., Renna M., Bellezza Oddon S., Rezaei Far A., Naser El Deen S., Veldkamp T. (2023). Insect meals in a circular economy and applications in monogastric diets. Anim. Front..

[bib0020] Gungor E., Erener G. (2020). Effect of dietary raw and fermented sour cherry kernel (*Prunus cerasus* L.) on digestibility, intestinal morphology and caecal microflora in broiler chickens. Poult. Sci..

[bib0021] Han J., Ullah M., Andoh V., Khan M.N., Feng Y., Guo Z., Chen H. (2024). Engineering bacterial chitinases for industrial application: from protein engineering to bacterial strains mutation! A comprehensive review of physical, molecular, and computational approaches. J. Agric. Food Chem..

[bib0022] Hatab M.H., Ibrahim N.S., Sayed W.A.A., Badran A.M.M., Rumpold B.A. (2025). Impact of Mediterranean fruit fly rearing residues and biological supplementation on performance of Gimmizah chicks. Poult. Sci..

[bib0023] Hwangbo J., Hong E.C., Jang A., Kang H.K., Oh J.S., Kim B.W., Park B.S. (2009). Utilization of house fly-maggots, a feed supplement in the production of broiler chickens. J. Environ. Biol..

[bib0024] Jha R., Mishra P. (2021). Dietary fiber in poultry nutrition and their effects on nutrient utilization, performance, gut health, and on the environment: a review. J. Anim. Sci. Biotechnol..

[bib0025] Kawasaki K., Zhao J., Sharma B., Khatun A., Sultana S., Kawasaki T., Ishikawa M., Ban T., Yano K. (2026). Nutritional evaluation of housefly larvae meal in broilers: growth performance, gut health, metabolic energy, and microbiota composition. Animals (Basel).

[bib0026] Kazemi M., Valizadeh R. (2025). Overcoming feed scarcity: how insects can transform sustainable animal nutrition. Cogent Food Agric..

[bib0027] Khan M., Chand N., Khan S., Naz S., Alrefaei A.F., Chandrasekaran A., Khan R.U. (2024). Partial replacement of soybean meal with *Musca domestica* larvae meal in broiler diets: implications for growth performance, nutrient utilization, hemato-biochemical profile and organoleptic characteristics. Arch. Anim. Breed..

[bib0028] Koh K., Iwamae S. (2013). Chitinolytic activity of mucosal enzymes in the different parts of the digestive tract in broilers. J. Poult. Sci..

[bib0029] Krysiak K., Konkol D., Korczyński M. (2021). Overview of the use of probiotics in poultry production. Animals (Basel).

[bib0031] Lee J., Park H., Heo J.M. (2025). Dietary additives to extend laying persistency in aged hens. Korean J. Poult. Sci..

[bib0030] Lee J.H., Kim T.K., Cha J.Y., Jang H.W., Yong H.I., Choi Y.S. (2022). How to develop strategies to use insects as animal feed: digestibility, functionality, safety, and regulation. J. Anim. Sci. Technol..

[bib0032] Liu Q., Liu Y., Shan C., Ma C., Li J., Jiang G. (2026). Probiotic-enzyme complex improves laying performance in laying hens fed low-protein diets by modulating gut microbiota and host metabolism. Front. Vet. Sci..

[bib0034] Mohammadi S., Torki M., Darbemamieh M. (2025). Effects of mealworm-based synbiotics on productive performance, egg quality, blood metabolites, and gut health in laying Japanese quails. Food Sci. Nutr..

[bib0035] Musigwa S., Cozannet P., Morgan N., Swick R.A., Wu S.B. (2021). Multi-carbohydrase effects on energy utilization depend on soluble non-starch polysaccharides-to-total non-starch polysaccharides in broiler diets. Poult. Sci..

[bib0036] Nayab G.E., Rashid S.B., Khan S., Sultan A., Ullah I., Khan R. (2025). Proximate composition and mineral analysis of housefly maggots and mealworms compared to conventional fish meal and soybean meal. BioRxiv.

[bib0037] Ochoa I., Valderrama E., Ayquipa E.M., Cárdenas L.A., Zea D., Huamani Z., Castellaro G. (2025). Productive yield, composition and nutritional value of housefly larva meal reared in high-altitude Andean zones of Peru. Animals (Basel).

[bib0038] Oketch E.O., Heo J.M. (2026). Prospects of feed additive incorporation in laying hen diets: a narrative review of principal biological effects and recent developments. J. Anim. Sci. Technol..

[bib0039] Pascon G., Opere Akinyi R., Cardinaletti G., Daniso E., Messina M., Tulli F. (2025). Chitin and its effects when included in aquafeed. Aquac. Int..

[bib0040] Penazzi L., Schiavone A., Russo N., Nery J., Valle E., Madrid J., Martinez S., Hernandez F., Pagani E., Ala U., Prola L. (2021). *In vivo* and *in vitro* digestibility of an extruded complete dog food containing black soldier fly (*Hermetia illucens*) larvae meal as protein source. Front. Vet. Sci..

[bib0041] Pieterse E., Pretorius Q. (2014). Nutritional evaluation of dried larvae and pupae meal of the housefly (*Musca domestica*) using chemical- and broiler-based biological assays. Anim. Prod. Sci..

[bib0042] Pretorius, Q. 2011. The evaluation of larvae of *Musca domestica* (common house fly) as protein source for broiler production. Doctoral diss. Stellenbosch Univ., Stellenbosch, South Africa.

[bib0043] Qiang T., Zhang K., Zeng Q., Ding X., Bai S., Liu Y., Xu S., Xuan Y., Li S., Wang J. (2025). Comparative analysis of intestinal and reproductive function in older laying hens with three egg laying levels. Front. Microbiol..

[bib0044] Rahayu S., Widiyastuti T., Suryapratama W., Hartoyo B., Rimbawanto E.A. (2023). Performance and feed digestibility of Sentul chicken fed hydrolyzed maggot (*Hermetia illucens*) meal produced by crude enzymes from Tempeh yeast. Afr. J. Food Agric. Nutr. Dev..

[bib0045] Rangel F., Monteiro M., Santos R.A., Ferreira-Martins D., Cortinhas R., Gasco L., Gai F., Pousão-Ferreira P., Couto A., Oliva-Teles A., Serra C.R. (2024). Novel chitinolytic *Bacillus* spp. Increase feed efficiency, feed digestibility, and survivability to *vibrio anguillarum* in European seabass fed with diets containing *Hermetia illucens* larvae meal. Aquaculture.

[bib0046] Salahuddin M., Abdel-Wareth A.A.A., Hiramatsu K., Tomberlin J.K., Luza D., Lohakare J. (2024). Flight toward sustainability in poultry nutrition with black soldier fly larvae. Animals (Basel).

[bib0047] Salehizadeh A., Torki M., Darbemamieh M., Sharifi S.D. (2025). Effects of housefly larvae meal and multi-enzyme-probiotic supplementation on performance, egg quality, and blood biochemistry in laying hens. Appl. Food Res..

[bib0048] Salehizadeh A., Torki M., Darbemamieh M., Sharifi S.D. (2025). Diet inclusion of housefly larvae and frass supplemented by Rayabold (enzyme and probiotic) on performance of laying hens and egg quality. Poult. Sci..

[bib0049] SAS (2015). SAS User's Guide.

[bib0050] Sedgh-Gooya S., Torki M., Darbemamieh M., Khamisabadi H., Abdolmohamadi A. (2021). Effect of dietary inclusion of yellow mealworm (*Tenebrio molitor*) larvae meal on productive performance, egg quality indices and blood parameters of laying hens. Anim. Prod. Sci..

[bib0051] Sekh N., Karki D. (2022). Dietary fiber in poultry nutrition in the light of past, present, and future research perspective: a review. Open J. Anim. Sci..

[bib0052] Tóth M., Birolo M., Palumbo B., Nalon E., Frangipane di Regalbono A., Giaccone V., Erdélyi M., Dalle Zotte A. (2025). Effect of insect farming by-product (frass) as a novel bedding material on litter quality, growth performance, carcass traits, and meat quality of broiler chickens. Front. Anim. Sci..

[bib0053] Yalveh B., Torki M., Darbemamieh M., Cheghamirza K., Sharifi R. (2026). Effects of *Tenebrio molitor* meal supplemented with *Saccharomyces cerevisiae* and/or *Bacillus velezensis*on production performance and egg quality in laying hens. Poult. Sci..

[bib0054] Yang Y., Xu S., Gao W., Wei Y., Wang L., Lai C. (2025). Chitinase improves the available energy, amino acids digestibility of black soldier fly and fecal microbiota of growing pigs. Anim. Biosci..

[bib0055] Yaqoob M.U., Yousaf M., Iftikhar M., Hassan S., Wang G., Imran S., Zahid M.U., Iqbal W., Wang M. (2022). Effect of multi-enzymes supplementation on growth performance, meat quality, ileal digestibility, digestive enzyme activity and caecal microbiota in broilers fed low-metabolizable energy diet. Anim. Biosci..

[bib0056] Yi W., Huang Q., Liu Y., Fu S., Shan T. (2024). Effects of dietary multienzymes on the growth performance, digestive enzyme activity, nutrient digestibility, excreta noxious gas emission, and nutrient transporter gene expression in white feather broilers. J. Anim. Sci..

[bib0057] Yildirim-Aksoy M., Eljack R., Beck B.H. (2020). Nutritional value of frass from black soldier fly larvae, *Hermetia illucens*, in a channel catfish. *Ictalurus Punctatus*, Diet. *Aquac. Nutr*.

[bib0058] Zhang J.C., Chen P., Zhang C., Khalil M.M., Zhang N.Y., Qi D.S., Wang Y.W., Sun L.H. (2020). Yeast culture promotes the production of aged laying hens by improving intestinal digestive enzyme activities and the intestinal health status. Poult. Sci..

[bib0059] Zou J., Zheng P., Zhang K., Ding X., Bai S. (2013). Effects of exogenous enzymes and dietary energy on performance and digestive physiology of broilers. J. Anim. Sci. Biotechnol..

[bib0060] Zuidhof M.J., Molnar C.L., Morley F.M., Wray T.L., Robinson F.E., Khan B.A., Al-Ani L., Goonewardene L.A. (2003). Nutritive value of house fly (*Musca domestica*) larvae as a feed supplement for turkey poults. Anim. Feed Sci. Technol..

